# Cellulose Pulp- and Castor Oil-Based Polyurethanes for Lubricating Applications: Influence of *Streptomyces* Action on Barley and Wheat Straws

**DOI:** 10.3390/polym12122822

**Published:** 2020-11-27

**Authors:** Antonio M. Borrero-López, Concepción Valencia, Alba Blánquez, Manuel Hernández, María E. Eugenio, José M. Franco

**Affiliations:** 1 Pro2TecS—Chemical Process and Product Technology Research Centre, Departamento de Ingeniería Química, ETSI, Campus de “El Carmen”, Universidad de Huelva, 21071 Huelva, Spain; am.borrero@diq.uhu.es (A.M.B.-L.); franco@uhu.es (J.M.F.); 2 Departamento de Biomedicina y Biotecnología, Universidad de Alcalá, 28805 Alcalá de Henares, Spain; alba.blanquez@edu.uah.es (A.B.); manuel.hernandez@uah.es (M.H.); 3 Forest Research Centre, Forest Products Department, INIA, 28040 Madrid, Spain; mariaeugenia@inia.es

**Keywords:** cellulose pulp, lubricating grease, oleogel, polyurethane, rheology, solid-state fermentation, *Streptomyces*, tribology

## Abstract

The replacement of mineral oils and non-renewable gelling agents is an imperative requirement for the lubricant industry in the near future. In this framework, cellulose pulp and castor oil are proposed as sustainable substitutes for these components. Biological treatment has been explored and evaluated to enhance the dispersing and thickening properties of cellulose pulp in oil media. *Streptomyces* sp. MDG147 and MDG301 strains were employed to modify agricultural wheat and barley straw residues from which cellulose pulp was obtained afterwards. In addition, an environmentally friendly process for the production of cellulose-pulp-/castor-oil-based polyurethanes was applied, in which neither catalysts nor harmful solvents were used, resulting in chemical oleogels. These oleogels were rheologically and tribologically characterized to evaluate their performance as lubricating greases. The enzymatic activity pattern developed was dependent on the raw material, the strain type, and the temperature, influencing the cellulose pulp’s composition, polymerization degree, and crystallinity. These modified characteristics tuned the rheological behavior of the different oleogels, providing a beneficial range of viscoelastic responses and viscosity values that were generally favored by the *Streptomyces* action. Furthermore, the friction coefficient and dimensions of wear scars measured in a tribological contact were comparable to, or even lower than, those found with commercial and other bio-based lubricating greases that have previously been studied.

## 1. Introduction

Current research is being driven by the mandatory necessity of finding new materials and proceedings that will enable harmful industrial processes, end-use products, and non-renewable energy sources to be replaced by greener alternatives, such as natural, renewable, or bio-based products and non-hazardous techniques that meet the trends of Green Chemistry [1].

Among the most studied bioresources, cellulose is undoubtedly the principal one. As a consequence of being the most abundant biopolymer on Earth, research has focused on many alternative fields, such as composites [2], tissue regeneration [3], chemical products [4,5], biosensors [6], energy storage [7], etc. Such diverse applications are possible because of cellulose’s main chemical and physical properties, which make of it an outstanding biopolymer. The linear chemical structure—based on glucose and linked by β(1–4) glycosidic bonds—and the numerous hydroxyl groups that allow the formation of a crystalline structure through hydrogen bonding [8] are some of its most interesting features. The fibrous structure and ability to form macromolecular networks have prompted the possibility of using cellulose to produce different gel-like structures, such as aerogels [9,10], hydrogels [11,12], or oleogels [13,14,15], for a wide variety of applications.

The development of bio-based oleogels may represent a new market niche in the lubricant industry with interesting perspectives for the future, since metallic soap-based thickening agents and mineral oils, the main components in lubricating grease formulations, may have to be replaced soon as a consequence of more restrictive politics [16,17]. At the Chemical Process and Product Technology Research Center (Pro2TecS, Universidad de Huelva), we have dedicated our efforts over the last two decades to the development of eco-friendly oleogels, attempting to provide them with outstanding functional properties so they can be proposed as efficient alternatives to traditional lubricating greases, as well as to the comprehensive characterization of the rheological and tribological behaviors of both commercial and bio-based lubricating greases [18,19]. More specifically, this research has been focused on the finding of suitable and sustainable gelling or thickening agents in oily media by following diverse approaches. Among the different raw materials, biopolymers such as chitosan and chitin [20], lignin [16,21,22,23], cellulose derivatives [14], and more complex lignocellulosic materials [17,24,25] have been used to thicken or gelify vegetable oils. Furthermore, chemical modifications, such as epoxidation [24], methylation [26,27], ethylation [28], acylation [20], or polyurethane formation [29], were applied to either decrease the polarity of these biopolymers—thus increasing the affinity by the oil medium—or promote the formation of chemical gels and more stable networks by generating covalent bonds between the biopolymer and the vegetable oil. Nonetheless, despite the fact that these final formulations can be considered bio-based, inert, and non-toxic materials, some of these procedures involve the use of hazardous chemicals and solvents; therefore, alternative processes and methodologies must be further explored. This is the case, for instance, of polyurethane-based materials, which are usually synthesized using toluene or other harmful solvents [30], as well as triethylamine or dibutyltindilaurate as catalysts [31]. Thus, procedures free of both catalysts and solvents have lately been focused upon [29].

On the other hand, bio-source modification has also widely been targeted in order to provide systems with better properties for certain applications, rather than the consequent use of harmful chemicals [32]. To overcome the challenge, biological treatments have been acutely pointed out as a greener alternative to chemical modifications. Fungi [33,34] and bacteria [16,35,36,37] are among the most used precursors, and are capable of altering the biomass through a degradation process. Some actinobacteria, like the genus *Streptomyces*, produce cellulolytic, hemicellulolytic, and ligninolytic enzymes, which break the main linkages that join cellulose, hemicellulose, and lignin, respectively [38]. The resulting bioproducts may achieve better characteristics than the non-modified ones, since this linkage breakdown is known to increase the content of functional groups, thus facilitating further reactions and altering the original structural features, which may favor the formation of more appropriate oleogels, as demonstrated in previous work on lignin-enriched streams [16]. Following this research line, the preparation of polyurethane oleogels with lubricating grease properties based on cellulose pulp and castor oil was targeted in this work. Several cellulose pulp samples were obtained from barley and wheat straws after being subjected to biological modification (solid-state fermentation) through the action of the *Streptomyces* sp. MDG147 and MDG301 strains. Afterwards, chemical oleogels were prepared by directly mixing the castor oil, cellulose pulp, and the diisocyanate crosslinker at room temperature, avoiding the use of harmful catalysts, solvents, and energy-consuming processes. Finally, the effect of the biological treatment on the rheological and some performance properties was evaluated.

## 2. Materials and Methods

### 2.1. Materials

Wheat (*Triticum aestivum* var. *maestro*) and barley (*Hordeum vulgare*) straws were kindly provided by farmers from Guadalajara (Spain). Hexamethylene diisocyanate (HDI) (>98% purity) was purchased from Merck (Darmstadt, Germany). Castor oil, with fatty acid contents of 5.3%, 7.0%, and 82.5% of oleic, linoleic, and ricinoleic acids, respectively, was supplied by Guinama (Valencia, Spain). All other common solvents and reagents used to perform the solid-state fermentation and soda pulping were acquired from Merck.

### 2.2. Solid-State Fermentation

Wheat and barley straws were submitted to solid-state fermentation (SSF) treatments by selecting two different types of *Streptomyces* sp. MDG147 and MDG301 strains. The first one finds its optimum working temperature at 28 °C, while the latter is considered a thermophilic strain, reaching an optimum activity at 45 °C. MDG301 activity was tested at 45 °C on both wheat and barley straws (samples CPW301.45 and CPB301.45) and 28 °C on wheat straw (sample CPW301), whereas MDG147 activity was evaluated only on barley straw (CPB147). More detailed information about preinoculum acquisition, SSF process, and enzymatic activity monitoring system can be found elsewhere [39]. Uninoculated samples followed a similar treatment for further comparison.

### 2.3. Soda Pulping

Once SSF was accomplished, a soda pulping process was carried out to separate both holocellulosic and lignin-enriched phases. This process has been thoroughly described in previous work [16]. Uninoculated wheat and barley straws were also submitted to soda pulping and were taken as references. Finally, large cellulosic pulp clusters obtained after soda pulping were milled by using a rotary miller MF 10 basic WERKE (IKA, Staufen, Germany) equipped with a 0.25 mm mesh.

### 2.4. Oleogel Processing

A green and straightforward procedure to prepare gel-like polyurethanes, which avoids the use of solvents and catalysts, was followed [29]. In brief, cellulose pulps were directly blended with HDI (1/2 cellulose pulp/HDI weight ratio) and castor oil (85% or 90% w/w) under stirring at room temperature (~20 °C) for 24 h using an RW20 (IKA, Staufen, Germany) equipped with an anchor impeller at 70 rpm.

### 2.5. Characterization Techniques

Cellulose pulp composition was determined by following the National Renewable Energy Laboratory’s standard analytical methods (NREL/TP-510-42618). Samples were subjected to acid hydrolysis to determine the carbohydrate composition. The liquids resulting from the hydrolysis were then analyzed to determine the sugar contents using high-performance liquid chromatography (1260 HPLC, Agilent, Waldbronn, Germany) fitted with a G1362A refractive index detector (Agilent, Waldbronn, Germany) and equipped with an Hi-PlexPb column (Agilent, Waldbronn, Germany) operated at 70 °C. Ultrapure water (A10 Milli-Q, Millipore, Burlington, MA, USA) was used as a mobile phase, and was pumped at a rate of 0.6 mL min^−1^. UV -Visible spectrophotometry (Jasco V-500, Jasco, Japan) at 205 nm was used to quantify acid-soluble lignin. The solid residue obtained after the acid hydrolysis was considered acid-insoluble lignin (called Klason lignin). Extractives and ashes of the samples were analyzed according to the NREL/TP-510-42619 and UNE 57050:2003 standards, respectively. Intrinsic viscosity [*η*] was calculated by following the ISO/FDIS 5351:2010 standard, from which the polymerization degree (DP) was obtained by applying the following equations indicated in the SCAN-CM 15:88 standard:

(1) $$DP=\frac{\left[\eta \right]}{0.42}\;for\;DP<950\;DP^{0.76}=\frac{\left[\eta \right]}{2.28}\;for\;DP>950$$

Thermogravimetric analysis (TGA) was performed by applying a ramp increasing at 10 °C/min from room temperature up to 600 °C in a Q-50 apparatus (TA Instrument Waters, New Castle, DE, USA), while Fourier transform infrared (FTIR) spectra were collected in a JASCO FTIR 4200 spectrometer (Jasco Inc., Tokyo, Japan) with wavenumbers from 400 to 4000 cm^−1^ and with a 4 cm^−1^ resolution.

Both linear viscoelasticity and viscous flow tests were performed in a controlled stress rheometer, MARS (Thermo Scientific, Darmstadt Germany), using a serrated plate–plate geometry (20 mm diameter and 1 mm gap). Frequency sweeps were carried out from 100 to 0.03 rad/s within the linear viscoelastic range, which was previously estimated by performing stress sweeps at 1 Hz, whereas viscous flow curves were obtained by applying an increasing stepped shear rate ramp within the 0.01–100 s^−1^ range.

Friction coefficient values were obtained in a Physica MCR-501 rheometer (Anton Paar, Graz, Austria) equipped with a tribological cell, consisting of a 6.35 mm diameter steel ball spinning on three 45°-inclined rectangular-shaped steel plates, on which the oleogel specimens acting as lubricants were spread. A constant normal load and a rotational speed of 20 N and 10 min^−1^, respectively, were applied for 10 min. This time was long enough to achieve stationary values of the friction coefficient. Five replicates were performed for each oleogel sample. The wear scars thereby produced in the steel plates were analyzed through optical microscopy using an Olympus microscope, BX51 model (Tokyo, Japan), from which both the parallel-to-the-rotation diameter and the typically elliptical wear area were determined. The data supplied here represent the mean of the three plates.

## 3. Results

### 3.1. Cellulose Pulp Composition

**Figure 1:**
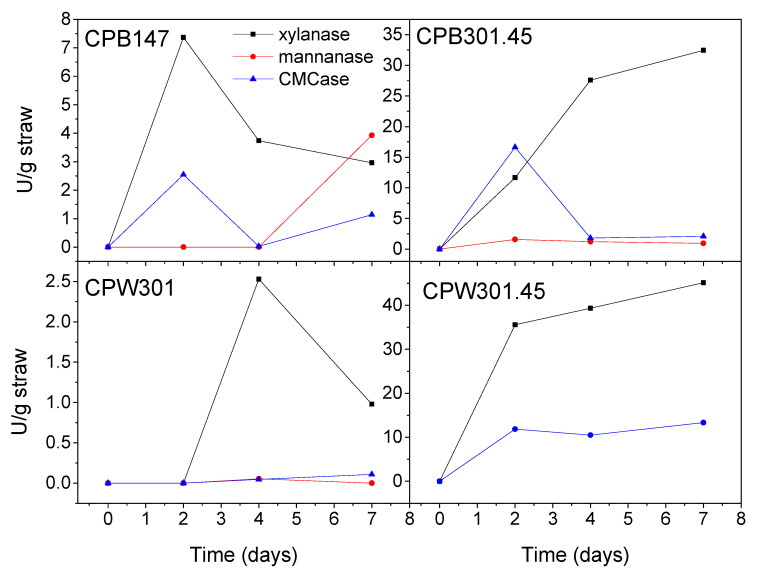
Enzymatic profiles for both wheat and barley straws treated under different conditions in the solid-state fermentation.

The composition of the different cellulose pulps obtained from barley and wheat straws, whether or not submitted to bacterial action in solid-state fermentation, is included in Table 1. In general, these results are supported by the enzymatic activity pattern of the diverse *Streptomyces* strains shown in Figure 1. This figure only displays the enzymatic activity of CMCase and hemicellulases, as laccases and peroxidases are known to remain attached to the substrate, which does not allow their separation for quantification [40,41]. These enzymatic profiles accurately reflect hemicellulose’s typical structure [42], as hemicellulase activities suggest a higher concentration of xylan units compared to mannans.

In barley-straw-derived cellulose pulps, it can be seen how the xylose concentration in systems previously fermented with MDG301 (CPB301.45) and MDG147 (CPB147) is slightly lower in comparison to the uninoculated one (CPB), which reveals the xylanase activity of these strains (see Figure 1). However, although the final xylanase concentration was more than 10 times higher for MDG301 compared to MDG147, xylose concentrations in the corresponding cellulose pulps did not differ significantly. This fact can be explained by also taking into account the higher activity of CMCase, which balances both hemicellulose and cellulose losses. As a consequence, the glucose concentration in cellulose pulps was generally increased by enzymatic action, and this increment was higher in the case of the MDG147 strain due to the lower CMCase production during SSF. Regarding the lignin content, similar concentrations for CPB (uninoculated) and CPB147 were observed. However, this content increased due to the MDG301 action, which may have been a consequence of a lower laccase production compared to the other enzymes.

In the case of wheat straw, only MDG301 was considered; nonetheless, this strain was tested at optimum (45 °C, cellulose pulp sample CPW301.45) and non-optimum conditions (28 °C, cellulose pulp sample CPW301). The enzymatic profile shown in Figure 1 allows the confirmation of these non-optimum conditions, as xylanase only achieved less than 10% activity on the fourth day and around 2% on the seventh day compared to the optimum conditions. Moreover, slightly lower glucose content and higher xylose content were respectively obtained in sample CPW301.45 in comparison with CPW301 as a consequence of the higher CMCase/xylanase enzymatic ratio. In any case, both the glucose and xylose concentrations in the cellulose pulps obtained from wheat straw submitted to SSF significantly differ from those obtained from the uninoculated wheat straw (CPW). Moreover, these results were not similar to those observed for barley straw, highlighting the importance of the bioresource. For instance, acting on wheat straw, at optimum conditions, MDG301 was able to deeply degrade lignin’s structure, reaching around 60% concentration with respect to the reference content, i.e., the uninoculated system, while the application of non-optimum temperature only exerted a slight influence on the soluble lignin content. On the other hand, CMCase and xylanase activities obtained at optimum temperature were much higher than those observed for barley straw. Nonetheless, despite these remarkable activities, the overall concentrations of glucose and xylose increased with respect to the reference system due to a more important loss of alternative extractives and both Klason and soluble lignin occurring simultaneously. The different responses of diverse enzymatic strains in similar bio-sources have already been reported for endoglucanases [43].

### 3.2. TGA and FTIR Spectroscopy of Cellulose Pulps

**Figure 2:**
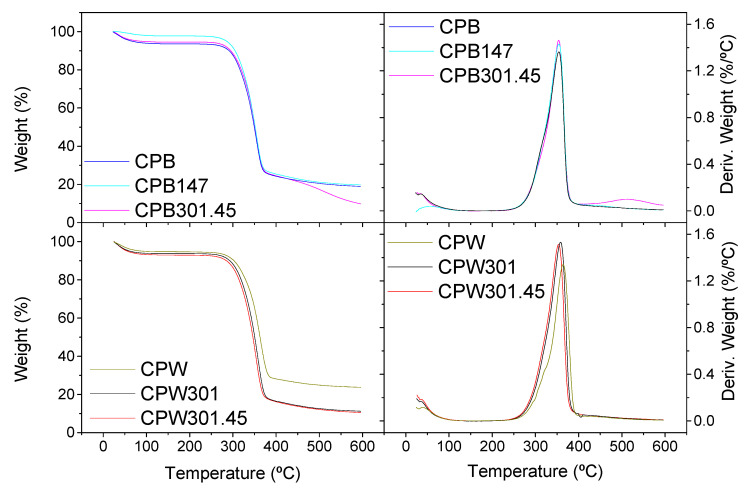
TGA curves, expressed as both the weight loss percentage and the associated derivative function, of (**top**) barley- and (**bottom**) wheat-straw-derived cellulose pulps.

The main characteristic parameters of cellulose pulps’ thermal degradation patterns—obtained from the TGA curves depicted in Figure 2—are presented in Table 2, i.e., the temperatures at which degradation steps begin (*T*_onset_) and end (*T*_final_), the temperature for the maximum degradation rate (*T*_max_), the weight losses (ΔW), and the final residue values.

Unlike the differences reported for straws submitted to SSF treatments with *Streptomyces* and the corresponding lignin fractions obtained [16,33], the TGA profiles of cellulose pulps were very similar and not very influenced by the SSF treatments. Thus, all of them exhibited an initial water loss of around 5–7% and a similar prominent main degradation event centered at around 354–364 °C as a consequence of the cellulose chain breakage [44]; however, some remarks may be pointed out. Both CPB and CPB147 cellulose pulps demonstrated similar final residue values of around 19–20%. Nonetheless, a deeper modification was again noticed due to MDG301 action, since an additional weight-loss stage can be detected at around 515 °C, finally reaching only 10% residue. This means that MDG301 action favors the degradation of initially stronger chemical structures, as the higher enzymatic activity can lead to the breakage of more hydrogen bonds, finally making it easier to thermally degrade cellulose [43]. On the other hand, for cellulose pulps obtained from wheat straw, very similar TGA profiles were found once submitted to SSF treatments with MDG301, regardless of the application of optimum or non-optimum temperatures, with residues of around 11% despite the already-discussed differences observed in the enzymatic patterns. On the contrary, cellulose pulp obtained from uninoculated wheat straw (CPW) exhibited higher *T*_max_ and residue values. These results allow us to conclude that, even though the enzymatic activity at non-optimum temperature was much smaller, it enables the removal of more easily thermally degradable products, as mentioned above, suggesting similar preferential targets regardless of the temperature.

**Figure 3:**
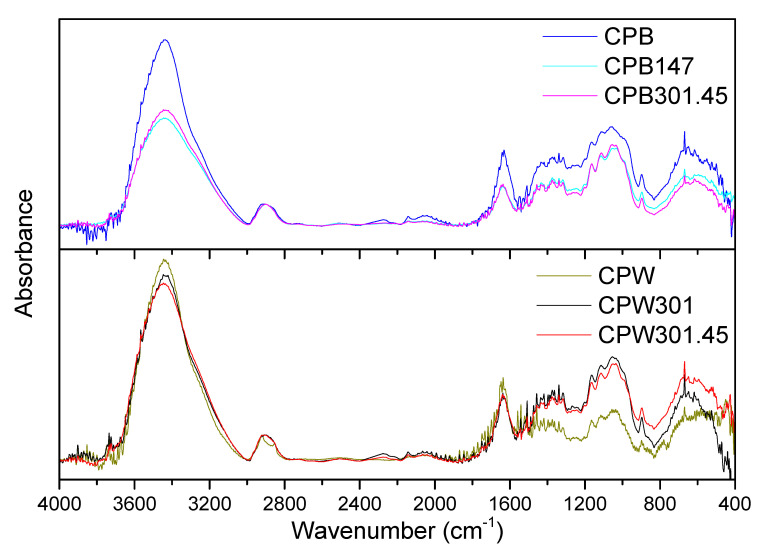
Fourier transform infrared (FTIR) spectra of (**top**) barley- and (**bottom**) wheat-straw-based pulps.

The FTIR spectra of all the cellulose pulps studied were also very similar (see Figure 3), as the compositions were not excessively different. However, as mentioned in the introduction section, cellulose pulps may show a certain degree of crystallinity, a consequence of hydrogen bonding, which can be addressed by evaluating the peaks centered around 1430 and 893 cm^−1^ [45]. These two peaks are due to symmetric CH_2_ bending vibration and C–O–C units of glucose, respectively, which are considered markers of crystallinity and amorphous behavior, respectively. Hence, the absorbance ratio between both peaks (A_1430_/A_893_) is considered an index to evaluate the relative crystallinity of different samples (Table 3). Therefore, higher relative crystallinity indices were obtained for cellulose pulp samples obtained from fermented barley straw in comparison to that obtained from the uninoculated one. This fact may be a consequence of the higher cellulose content and more hydroxyl groups available, which may induce hydrogen bonding. Nonetheless, the opposite behavior was, once again, observed for cellulose pulps obtained from wheat straw, where fermented samples exhibited a clear reduction in the relative crystallinity index, which is probably due to the extreme levels of the MDG301 enzymatic activity compared to those measured in barley straw (see Figure 1), then leading to very degraded systems that were not able to generate crystalline structures to such a degree, i.e., breaking of hydrogen bonding [43].

It is also well known that FTIR bands are shifted to lower wavenumbers when stronger interactions are formed; thereby, the strength of hydrogen bonding within the cellulose structure can also be evaluated using FTIR spectra. Thus, the higher the shifting, the higher the energy of the linkage, as expressed in Equation (2) [46]:

(2) $$E_{H}=\frac{1}{K}\cdot \frac{\left(\upsilon_{0}-\upsilon \right)}{\upsilon_{0}}$$

where *v*_0_ and *v* are the initial wavenumber corresponding to the stretching absorption of –OH groups (3600 cm^−1^) and the wavenumber for –OH groups once SSF-induced structural changes have occurred, respectively, whereas *K* is a constant whose value is 4.02 × 10^−3^ kJ. In this case, the *E_H_* values are quite similar for most of the samples (see Table 3). Nonetheless, once again, the lowest energy was evinced by the CPW301-45 sample, supporting the higher degree of biological modification deduced from other techniques and analyses.

### 3.3. Polymerization Degree of Cellulose Pulps

The polymerization degrees of cellulose pulp samples estimated from Equation (1) have also been included in Table 3. In general, the MDG301 strain was able to produce cellulose pulps with a higher polymerization degree, especially when the strain worked at its optimum temperature. In principle, these results may be unexpected, but can be explained considering the degradation and possibly further elimination during pulping of numerous easily accessible structures and/or low-molecular-weight segments. This increment in DP has already been observed in other cellulose pulps where preferential removal of xylan was accomplished [47]. Actually, this fact results in higher proportions of stronger networks and longer cellulose chains, leading to an increase in intrinsic viscosity. Nonetheless, the MDG147 strain did not exert any influence on this parameter.

### 3.4. Rheology of Cellulose-Pulp-Based Oleogels

**Figure 4:**
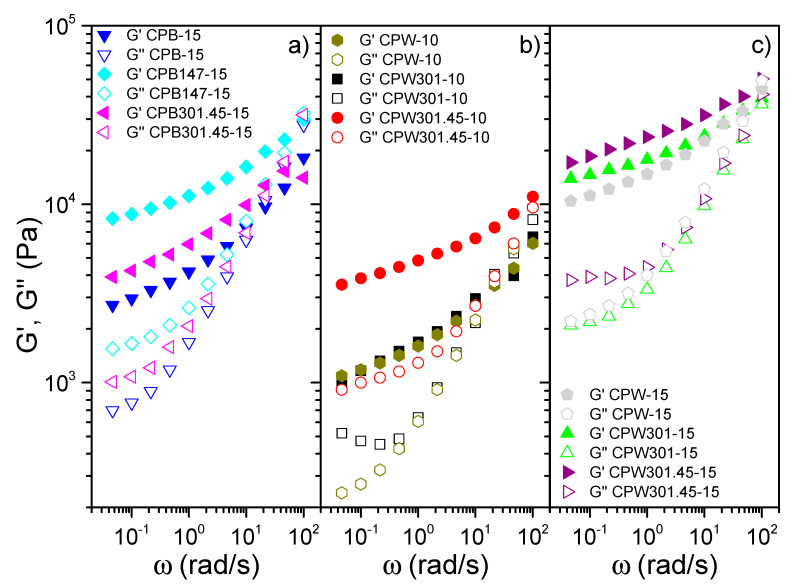
Evolution of the storage and loss moduli with frequency for oleogels containing (**a**) 15% barley-straw-derived cellulose-pulp-based thickener, (**b**) 10% wheat-straw-derived cellulose-pulp-based thickener, and (**c**) 15% wheat-straw-derived cellulose-pulp-based thickener.

Polyurethane oleogels were obtained by simply mixing the different cellulose pulps obtained from barley and wheat straws and HDI in the castor oil medium, as detailed for lignin-based polyurethanes [29]. Using 10–15% *w*/*w* of thickener agent (cellulose pulp and HDI at 1/2 weight ratio), the typical gel-like response of lubricating greases was achieved, covering a relatively wide range of values for the linear viscoelastic functions [19,48], as can be seen in Figure 4. Although the frequency dependence of the small-amplitude oscillatory shear (SAOS) functions was similar in all cases, in general, higher values of both the storage, G’, and the loss, G”, moduli were found when the cellulose pulps from fermented straws were used. For cellulose pulps derived from barley straw (Figure 4a), higher increments of the SAOS functions were observed when the straw was modified with the strain MDG147 (sample CPB147-15) in comparison with that modified with MDG301 (sample CPB301.45-15). This result is a consequence of the synergetic effect of both the higher cellulose content caused by the lower CMCase production and the lower lignin content, which may be due to a higher laccase production. These two opposite effects caused by cellulose and lignin concentrations have already been reported to modulate the viscoelastic response of non-modified cellulose pulp dispersions in vegetable oils [25].

Regarding the cellulose pulps derived from wheat straw, the slight rheological modification achieved in the polyurethane oleogels by the action of MDG301 at non-optimum temperature can be clearly observed for both thickener percentages (Figure 4b,c), as the viscoelastic response is almost identical to that obtained with the uninoculated wheat-straw-derived cellulose pulp. On the contrary, the action of the MDG301 strain at its optimum temperature (sample CPW301.45) was able to produce a noticeable increment in the SAOS functions, especially when using a 10% thickener concentration. Nonetheless, this difference is dampened to some extent at higher concentration, viz. 15%, due to the higher diisocyanate content, which can mask the biopolymer influence, as already found in previous work with lignin-based polyurethanes [22]. Again, the highest viscoelastic moduli were concomitant with the wheat-straw-derived cellulose pulps’ composition, as the highest glucose/lignin ratio was shown by the sample CPW301.45 compared to the other cellulose pulps obtained from wheat straw. Furthermore, as already demonstrated in a previous work regarding lignin-based oleogels [16], a higher enzymatic activity may somehow modify the lignocellulosic structure, making it more feasible for reaction with HDI by increasing the number of available hydroxyl groups, and thus yielding stronger crosslinked networks. In addition, along with the composition, a higher polymerization degree was also pointed out to significantly increase the viscoelastic functions of physically stabilized cellulose pulp dispersions [25]. The linear viscoelastic behavior of these bio-based oleogels is similar to that shown by commercial lithium lubricating greases, which traditionally exhibit G′ values of around 10^4^ Pa and G″ values around one order of magnitude lower, although a more extended plateau region within the frequency range studied is generally found [19].

**Figure 5:**
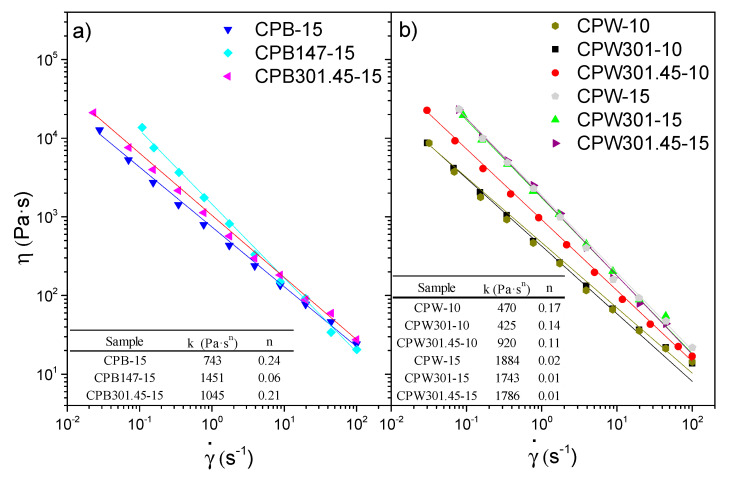
Viscous flow curves of polyurethane oleogels prepared with (**a**) barley- and (**b**) wheat-straw-derived cellulose pulps. Values of the consistency and flow indexes are also included as an inset.

Regarding the viscous flow behavior of these polyurethane oleogels, a markedly shear-thinning response was obtained in all cases, as shown in Figure 5, with a viscosity decay of several decades with the increasing shear rate, which is also a distinctive characteristic of traditional lubricating greases [49]. This power-law evolution, generally associated with very low values of the flow index, is, however, part of a more general and complex viscous flow behavior of this type of material (see, for instance, [18]). In fact, at very low shear rates, a tendency to achieve constant high viscosity values should be observed, although the controversy about the existence of an apparent yield stress value is still open, whereas at very high shear rates, again, constant values of limiting viscosity must be reached. However, reliable viscosity data above 100 s^−1^ are not easy to obtain in rotational rheometers as a consequence of different flow problems, like the fracture of the sample.

Similarly to what was discussed for the viscoelastic response, generally, the highest viscosity values were obtained for the oleogels prepared with cellulose pulps containing higher glucose/lignin ratios. Nonetheless, at high shear rates, this tendency may be dampened, or even reversed, since more structured oleogels tend to exhibit a stronger shear-thinning character, as observed for the CPB147-15 oleogel (Figure 5a). Finally, almost identical viscosity curves were obtained for all the polyurethane oleogels prepared with wheat-straw-derived cellulose-pulp-based thickeners at 15 wt.%, revealing once again that, at this concentration, the rheological response is mainly governed by the crosslinker.

In all cases, the power-law model fits the viscous flow behavior fairly well within the shear rate range studied:

(3) $$\eta =k\cdot {\dot{\gamma }}^{n-1}$$

where *k* and *n* are the consistency and flow indexes, respectively. The values of these fitting parameters are listed in the insets of Figure 5. As can be seen, extremely low values of the flow index, *n*, were always obtained, especially for the most structured systems, as discussed above, which is representative of the yielding behavior typically exhibited by lubricating greases [18]. Moreover, the *k* values of these oleogels are also close to those shown by commercial calcium and lithium lubricating greases and model polyolefin-thickened lithium greases, which exhibited values in the range of 600–1500 Pa·s^n^ [19,49], again highlighting the rheological similarity between the traditional products and the obtained ones. Finally, it is worth mentioning that the samples showing higher values of the viscoelastic functions also exhibit higher *k* values.

### 3.5. Tribological Performance

**Figure 6:**
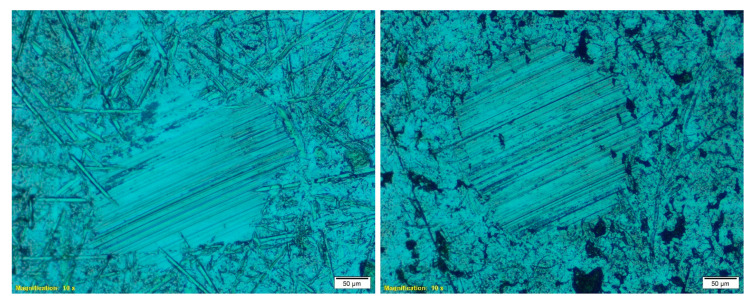
Selected pictures of wear marks produced during the tribological tests when using polyurethane oleogels prepared with barley- (CPB147-15) (**left**) or wheat- (CPW301.45-10) (**right**) straw-derived cellulose pulps as lubricants.

In order to test the lubrication performance of these cellulose-pulp-based polyurethane oleogels, friction and wear were assessed in a ball-on-three inclined plate steel–steel tribological contact [50] at both constant rotating speed and normal load. In all cases, highly satisfactory values of the friction coefficient were obtained, lower than those obtained when conventional greases [19] or previously functionalized cellulose-based oleogels [17] were used as lubricants. In general, the values of the friction coefficient tend to increase with the viscosity of the oleogel sample used as lubricant at high shear rates. Thus, for instance, very similar values of the friction coefficient were found for all the oleogels prepared with barley-straw-derived cellulose pulps, which presented almost identical viscosity values at high shear rates (see Figure 5a). On the contrary, cellulose pulp obtained from wheat straw submitted to SSF treatment with the MDG301 strain at its optimum temperature provided oleogels that produced slightly higher values of the friction coefficient in the tribological contact. As the oil medium was the same in all oleogel samples, these differences must be attributed to the thickener. Either the cellulose pulps containing higher cellulose pulp/lignin ratios—presumably yielding more crosslinked networks and also generally having higher DP values—favor oil entrapment, thus preventing its release, or simply the whole oleogel sample, including the thickener, can penetrate in the lubricating contact, and the highest friction is just a consequence of the higher viscosity [18]. Since the wear scars associated with the friction experiments were generally reduced when fermented-straw-derived cellulose pulps were used as a thickener agent, the second assumption seems to be more feasible. The observation of the wear scars generated during the friction tests (see some examples in Figure 6) allows us to conclude that the main wear mechanism was abrasion, supporting the idea that thickener particles penetrate into the mating surface, thus contributing to increased friction, especially at higher concentrations. These marks were evaluated in terms of both parallel-to-spin diameter and scar area (data shown in Table 4). As mentioned above, lower scar sizes were measured when cellulose pulps obtained from straw submitted to SSF treatments were employed, especially those resulting from the MDG301 action at its optimum temperature. Overall, outstanding wear values were obtained, lower than those found with commercial and other bio-based lubricating greases [51,52].

## 4. Conclusions

The modification of the lignocellulosic structures of both wheat and barley straw was accomplished through the action of the *Streptomyces* sp. MDG147 and MDG301 strains, also leading to alteration of the derived cellulosic pulps, which were further used to prepare polyurethane oleogels. This modification, as demonstrated by the lignocellulosic composition, is a reflection of the enzymatic activity pattern developed, which was also demonstrated to significantly affect the crystalline structure and polymerization degree of cellulose. The MDG301 strain caused more important modifications of the cellulose pulps, especially on those derived from wheat straw, when working at its optimum temperature, generally yielding a higher cellulose/lignin ratio and polymerization degree. These modifications had direct and significant impacts on the rheological and tribological behaviors of the polyurethane oleogels prepared with these cellulose pulps. SSF treatments to which the wheat and barley straws were previously subjected proved to be effective in enhancing the strength of the resulting oleogels, thereby increasing the values of both linear viscoelastic functions and viscosity. In addition, these oleogels showed outstanding frictional and wear behaviors when used as lubricants in a tribological contact, with similar or even lower values of both the friction coefficient and the size of resulting wear marks than those found in commercial lubricating greases. Overall, fermented-straw-derived cellulose pulps can be conclusively proposed as effective thickeners for preparing polyurethane oleogels with tunable properties depending on the biological modifications, as well as a potential substitute for non-renewable gelling agents in lubricating grease formulations.

## Figures and Tables

**Table 1:** Composition of the cellulose pulp samples.

Sample	Klason Lignin (%)	Soluble Lignin (%)	Glucose (%)	Xylose (%)	Arabinose (%)	Extracts (%)	Ashes (%)
**CPB**	2.2 ± 0.2	0.3 ± 0.1	62.2 ± 0.5	30.3 ± 0.5	3.2 ± 0.2	0.66	1.2 ± 0.2
**CPB147**	2.1 ± 0.3	0.3 ± 0.0	65.3 ± 0.8	28.1 ± 2.1	3.0 ± 0.2	0.44	1.3 ± 0.2
**CPB301.45**	3.5 ± 0.3	0.4 ± 0.0	64.2 ± 1.3	27.6 ± 0.7	3.1 ± 0.0	0.41	1.2 ± 0.0
**CPW**	6.3 ± 0.3	1.1 ± 0.1	55.5 ± 2.6	21.6 ± 1.8	1.1 ± 0.3	5.08	1.2 ± 0.2
**CPW301**	6.3 ± 0.6	0.3 ± 0.2	62.0 ± 2.2	29.7 ± 0.7	2.5 ± 0.0	1.14	0.6 ± 0.4
**CPW301.45**	4.0 ± 1.4	0.4 ± 0.0	61.2 ± 3.5	31.1 ± 3.7	4.3 ± 2.5	0.27	1.3 ± 0.3

**Table 2:** Characteristic thermogravimetric analysis (TGA) parameters of the cellulose pulps studied.

Sample	*T*_onset_ (°C)	*T*_max_ (°C)	*T*_final_ (°C)	ΔW (%)	Residue (%)
CPB	290	355	368	75	19
CPB147	288	354	368	78	20
CPB301.45	288/473	355/515	368/553	72/13	10
CPW	293	364	380	71	24
CPW301	291	358	373	82	11
CPW301.45	286	354	370	82	11

**Table 3:** Relative crystallinity index, energy related to hydrogen bonds (E_H_), and polymerization degree of the cellulose pulps studied.

	Relative Crystallinity Index	E_H_ (kJ)	Intrinsic Viscosity (cm^3^/g)	Polymerization Degree
CPB	1.24	11.31	494	1185
CPB147	1.29	11.18	492	1178
CPB301.45	1.51	11.31	700	1875
CPW	2.03	11.24	397	944
CPW301	1.45	11.31	544	1344
CPW301.45	1.10	10.78	608	1556

**Table 4:** Values of the friction coefficient and sizes of wear scars.

Oleogels	Friction Coefficient	Wear Scar Surface (µm^2^)	Wear Scar Diameter (µm)
CPB-15	0.081 ± 0.003	55,640 ± 2803	315 ± 32
CPB147-15	0.082 ± 0.003	41,024 ± 14,467	263 ± 69
CPB301.45-15	0.089 ± 0.006	28,551 ± 5918	190 ± 28
CPW-10	0.077 ± 0.003	35,669 ± 5704	217 ± 24
CPW301-10	0.077 ± 0.006	105,796 ± 7362	366 ± 2
CPW301.45-10	0.085 ± 0.006	63,314 ± 13,295	286 ± 23
CPW-15	0.099 ± 0.010	35,148 ± 504	215 ± 23
CPW301-15	0.088 ± 0.010	22,306 ± 11,749	161 ± 23
CPW301.45-15	0.108 ± 0.013	25,205 ± 3769	193 ± 30
